# Improving Social Connections Among Black Dementia Caregivers Through Community Engagement

**DOI:** 10.1093/geroni/igaf122.1286

**Published:** 2025-12-31

**Authors:** Sandhya Seshadri, Phyllis Jackson, Paula Alio, Sarah Morgan, Angela Contento, Benzi Kluger

**Affiliations:** University of Rochester, Rochester, New York, United States; Common Ground Health, Rochester, New York, United States; University of Rochester School of Medicine & Dentistry Department of Public Health, Rochester, New York, United States; University of Rochester, School of Medicine & Dentistry, Rochester, New York, United States; University of Rochester Warner School of Education, Rochester, New York, United States; University of Rochester, School of Medicine & Dentistry, Rochester, New York, United States

## Abstract

Limited access to resources, economic inequality, and cultural beliefs about dementia may lead to increased social isolation and loneliness among Black caregivers of persons living with dementia (PLwD). On the other hand, religious activities and spiritual connectedness are integral to Black caregiver wellbeing. To improve social connections among Black caregivers of PLwD we collaborated with a community advisory board (CAB) comprising members of the Interdenominational Health Ministry Coalition (IHMC), a local faith-based organization. The study was guided by the Dimensions of Social Connection Model to promote social connections in dementia caregivers (Van Orden & Heffner, 2022). The study aims were to (1) conduct semi-structured interviews with Black caregivers of PLwD to understand their caregiving experiences and social needs, and (2) co-develop the Congregational Compassionate Care (CCC) intervention. Our community-engagement practices were based on trust, respect, and mutual learning. Findings from the first aim informed the co-development of the six-week intervention that will be delivered by trained IHMC member-facilitators. The interactive Reach, Effectiveness, Adoption, Implementation, Maintenance (RE-AIM) toolkit guided CCC co-development. Researchers and CAB members held 5 workshops to co-develop the CCC intervention topics and content, a member-facilitator training program, resources for participants and volunteers, measures to assess feasibility, acceptability, social isolation, loneliness, and intervention fidelity, and manuals for participants and facilitators. In the next step we will collaboratively train member-facilitators and test the intervention. Our community engaged practices facilitated the development of a culturally relevant, community-delivered intervention that fostered community ownership of efforts to improve social connections among Black caregivers.

